# Sacubitril/Valsartan for Refractory Hypertension in Acute Intracerebral Hemorrhage: A Single-Center Retrospective Study

**DOI:** 10.7759/cureus.106155

**Published:** 2026-03-30

**Authors:** Koki Ito, Shinsuke Sato, Akitsugu Kawashima, Takakazu Kawamata, Yasunari Niimi

**Affiliations:** 1 Department of Neurosurgery, St. Luke’s International Hospital, Tokyo, JPN; 2 Department of Neurosurgery, Tokyo Women’s Medical University, Tokyo, JPN; 3 Department of Neuroendovascular Therapy, St. Luke’s International Hospital, Tokyo, JPN

**Keywords:** angiotensin receptor-neprilysin inhibitor, blood pressure, hemorrhagic stroke, intracerebral hemorrhage, sacubitril/valsartan, severe hypertension

## Abstract

Introduction: Intracerebral hemorrhage (ICH) is associated with high early mortality. Guidelines recommend a rapid and sustained reduction of systolic blood pressure to <140 mmHg. Achieving and maintaining this target in refractory hypertension often requires prolonged intravenous therapy and complex escalation of oral antihypertensive regimens. Sacubitril/valsartan, an angiotensin receptor-neprilysin inhibitor (ARNI), combines renin-angiotensin blockade with natriuretic peptide augmentation and has proven antihypertensive efficacy; however, data about its real-world use during the acute phase of ICH remain limited.

Methods: We performed a single-center retrospective cohort study of consecutive patients with acute ICH and untreated refractory hypertension. Following the institutional introduction of ARNI in 2022, patients receiving ARNI as a scheduled second-line oral antihypertensive were compared with historical controls. The primary endpoint was time to antihypertensive regimen stabilization, defined as the time point at which escalation of scheduled oral antihypertensives was no longer required and intravenous or rescue short-acting antihypertensives were discontinued. Secondary outcomes included the number of scheduled oral antihypertensives at stabilization and the discharge modified Rankin Scale (mRS) scores.

Results: Thirty patients with ARNI (ARNI group: 2022-2024) and 30 consecutive historical controls (non-ARNI group: pre-2021) were included. Baseline characteristics were similar between groups. The mean time to antihypertensive regimen stabilization tended to be shorter in the ARNI group, i.e., 119.6 hours in the ARNI group and 143.3 hours in the non-ARNI group (unadjusted p = 0.275). In multivariable linear regression, ARNI use was independently associated with shorter stabilization time (partial regression coefficients (B) = -40.427 hours; 95% confidence interval (CI): -79.833 to -1.021; p = 0.045). At stabilization, the ARNI group required fewer scheduled oral antihypertensives compared to controls (median: 2.0 (interquartile range: 2.0-2.0) vs. 2.0 (2.0-3.0); p = 0.030; adjusted B = -0.483; 95% CI: -0.825 to -0.140; p = 0.006). The discharge mRS scores did not differ between the groups.

Conclusions: In our routine clinical practice, early incorporation of ARNI as a second-line oral antihypertensive in acute ICH was associated with earlier completion of antihypertensive regimen adjustment processes and reduced treatment complexity. These results support the usefulness of ARNI as a practical second-line antihypertensive for goal-oriented blood pressure management and provide hypothesis-generating data for future prospective studies.

## Introduction

Spontaneous intracerebral hemorrhage (ICH) accounts for 10-15% of all strokes, is associated with high morbidity and mortality, and has an all-cause mortality of approximately 30-40% within 30 days of ICH onset [1-3]. Blood pressure elevation is a common finding in ICH patients because of a combination of mechanisms, including pre-existing hypertension, increased intracranial pressure, activation of the sympathetic pathway, and stress [4]. Strict blood pressure control significantly improves the functional outcomes in patients with ICH [5-7]. Nevertheless, this can be challenging, especially in patients with refractory hypertension, which often necessitates a prolonged infusion of intravenous antihypertensives and rescue short-acting oral antihypertensives [8]. Also, practical guidance regarding the optimal composition of oral antihypertensive regimens following initial intravenous therapy is limited. Consequently, the selection of second-line oral antihypertensive agents is frequently based on institutional preference rather than robust evidence. Sacubitril/valsartan sodium hydrate, a first-in-class angiotensin receptor-neprilysin inhibitor (ARNI) that blocks angiotensin II receptor-1 and neprilysin, exhibits potent and sustained antihypertensive effects [9]. Valsartan and sacubitril work together to enhance blood pressure control via complementary mechanisms. Specifically, valsartan blocks angiotensin II type 1 receptors, preventing angiotensin II-mediated vasoconstriction, while sacubitril increases the levels of natriuretic peptides (NPs) by preventing their breakdown [9]. NPs cause vasodilation, increase glomerular filtration, promote natriuresis and diuresis, and inhibit sympathetic and renin-angiotensin activities, resulting in a lower preload, systemic vascular resistance, and arterial pressure [10]. Additionally, sacubitril prevents angiotensin II degradation; nonetheless, the antihypertensive effect of ARNI is achieved through its interaction with valsartan and increased NPs [11]. ARNI has demonstrated superior blood pressure reduction compared to angiotensin II receptor blockers (ARB) alone. Notably, no previous studies have assessed ARNI as part of an acute antihypertensive strategy for ICH. Observing how blood pressure management has changed following the introduction of ARNI may provide useful insights into antihypertensive regimen management in this challenging population. Therefore, we conducted a retrospective cohort study to evaluate the introduction of ARNI as a scheduled second-line oral antihypertensive in patients with acute ICH and refractory hypertension.

## Materials and methods

Study design and setting

We performed a retrospective cohort study at St. Luke’s International Hospital, a tertiary care center in Japan. The study period spanned from April 2020 to December 2024. The study protocol was approved by the Institutional Review Board of the hospital (approval number: 24-R157), and the requirement for written informed consent was waived due to the retrospective nature of the study. At our institution, general consent for the use of clinical data for research purposes is obtained from patients, and those who declined participation were excluded from the study. The study adhered to the Declaration of Helsinki and institutional regulations. The STROBE (Strengthening the Reporting of Observational Studies in Epidemiology) guidelines were adhered to in this study.

We screened consecutive adult patients (≥18 years) admitted with spontaneous ICH. ARNI was first used for ICH in January 2022, and the use of ARNI has been the standard for ICH patients with refractory hypertension in our hospital since then. Patients treated after this date who received ARNI as a scheduled second-line oral antihypertensive constituted the ARNI group, whereas patients admitted prior to December 2021 who met the same clinical criteria served as historical controls (non-ARNI group). The inclusion criteria were as follows: (1) ICH with untreated hypertension or interruption of antihypertensives at onset; (2) requirement for at least two scheduled oral antihypertensive agents after administration; (3) initiation of the second-line oral antihypertensive within 48 hours of admission. Patients with secondary causes of hemorrhage other than hypertension, patients with intraventricular hemorrhage, patients who declined to participate, or who had a hospital stay ≤7 days were excluded. To focus on early antihypertensive decision-making, patients in whom the second-line agent was initiated on or after hospital day three were excluded. In this study, refractory hypertension was defined as blood pressure that could not be adequately controlled with a single scheduled oral antihypertensive agent during hospitalization, requiring the addition of a second agent.

Blood pressure management protocol and exposure definition

The institutional target systolic blood pressure (sBP) was less than 140 mmHg based on current guideline recommendations for acute ICH and standardized institutional protocol. Intravenous antihypertensive agents such as nicardipine and nitroglycerin were initiated immediately after diagnosis, and scheduled oral antihypertensives were added and increased as necessary while monitoring blood pressure. Intravenous antihypertensives were gradually tapered according to blood pressure control, with the aim of achieving stable blood pressure using scheduled oral antihypertensives alone. Rescue short-acting oral antihypertensives (nifedipine) were used as needed for transient blood pressure elevations. In the ARNI group, ARNI was used as the scheduled second-line oral antihypertensive. The initial ARNI dose was sacubitril/valsartan 49 mg/51 mg or 97 mg/103 mg once daily (total daily dose: 100 or 200 mg), up-titrated according to blood pressure control to a maximum of 194 mg/206 mg once daily (total daily dose: 400 mg). During the study period, the institutional blood pressure management protocol for acute ICH remained largely unchanged except for the introduction of ARNI as a second-line oral antihypertensive.

Outcomes

The primary outcome was “time to antihypertensive regimen stabilization”. This was defined as the interval from admission to completion of scheduled oral antihypertensives adjustment (completion of medication increase or addition) with discontinuation of intravenous and rescue short-acting antihypertensives. The secondary outcomes included the number of scheduled oral antihypertensive medications at stabilization and the modified Rankin Scale (mRS) score at discharge.

Statistical analysis

Continuous variables are reported as mean ± standard deviation or median (interquartile range) and compared by Student’s t-test or Mann-Whitney U test as appropriate; categorical variables are reported as counts (percentages) and compared with Fisher’s exact test. Multivariable linear regression was performed as an exploratory analysis to adjust for major clinical factors and to describe associations rather than to infer causality, with results interpreted descriptively. Because the outcome was observed in all patients during hospitalization without censoring, linear regression analysis was applied. Partial regression coefficients (B) with 95% confidence intervals (CIs) are presented. Model assumptions were assessed by inspection of residual plots, and no major violations were observed. All statistical analyses were performed using EZR version 1.68 (Jichi Medical University, Tochigi, Japan), which is a graphical user interface for R version 4.5.1 (The R Foundation for Statistical Computing, Vienna, Austria). More precisely, it is a modified version of R Commander designed to add statistical functions frequently used in biostatistics [12].

## Results

**Figure 1:**
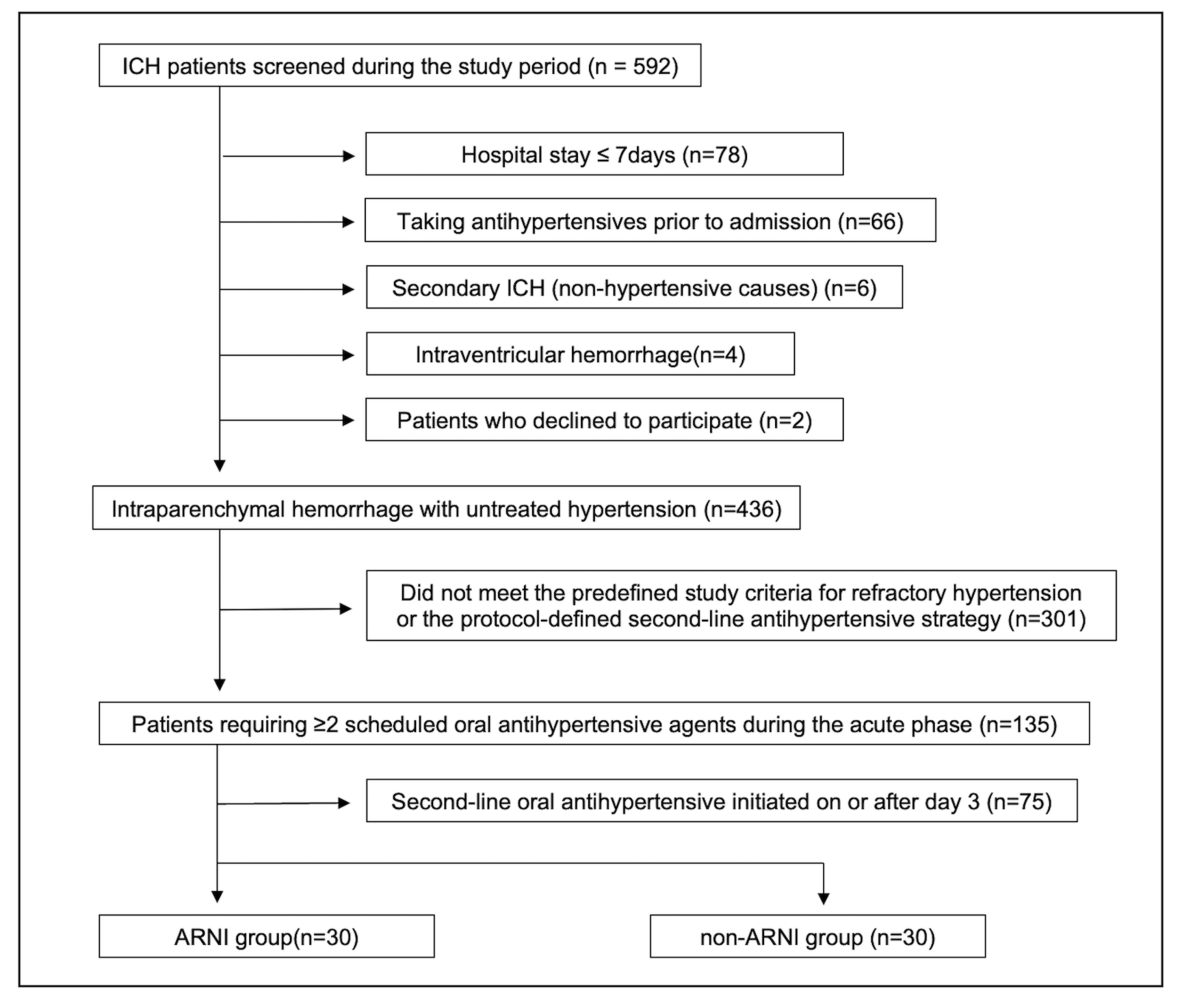
Flow diagram of patient selection. Patients with spontaneous ICH were screened during the study period. After exclusion of secondary causes, i.e., intraventricular hemorrhage, prior antihypertensive use, patients who declined to participate, and short hospital stay, patients with untreated refractory hypertension requiring ≥2 scheduled oral antihypertensives were identified. Patients who did not meet the predefined criteria for refractory hypertension or the study-defined second-line antihypertensive strategy were excluded. Patients were classified according to second-line oral antihypertensive strategy (ARNI vs. non-ARNI). Only patients in whom second-line therapy was initiated within two days were included in the final analysis. Day 0 = admission; ICH, intracerebral hemorrhage; ARNI, angiotensin receptor-neprilysin inhibitor.

Sixty patients met the inclusion criteria: 30 patients in the ARNI group (2022-2024) and 30 historical controls (pre-2021) (Figure 1). Table 1 summarizes the clinical and treatment characteristics of the patients. Baseline demographics and clinical severity were comparable between groups, including age, sex, admission blood pressure, Glasgow Coma Scale score, and hematoma volume. Treatment-related variables and safety outcomes, including surgical interventions, hematoma expansion, and antihypertensive-related adverse events (e.g., hypotension, acute kidney injury, and hyperkalemia), did not differ significantly. The duration and total dose of intravenous antihypertensive administration were also not significantly different between groups.

**Table 1:** Baseline characteristics, treatments, and outcomes. Values are median (IQR) unless otherwise indicated. Continuous variables with approximately normal distribution are presented as mean (SD). A p-value ≤ 0.05 (bold) was considered statistically significant. # Mann-Whitney U test; † Fisher's exact test; * Student’s t-test. Definitions. Hyperkalemia: serum potassium >5.5 mmol/L; acute kidney injury: increase in serum creatinine from baseline by ≥0.5 mg/dL or ≥150%. ARNI, angiotensin receptor-neprilysin inhibitor; IQR, interquartile range; sBP, systolic blood pressure; dBP, diastolic blood pressure; GCS, Glasgow Coma Scale; IV, intravenous; γ, μg/kg/min; ICU, intensive care unit; SD, standard deviation; mRS, modified Rankin Scale.

Variable	All (n = 60)	ARNI (n = 30)	Non-ARNI (n = 30)	P-value
Demographics				
Age, years, median (IQR)	56.0 (50.0-64.0)	54.0 (49.3-68.8)	57.0 (52.0-62.0)	0.63#
Female, n (%)	14 (23.3%)	6 (20%)	8 (26.7%)	0.76†
Clinical features				
sBP on admission, mmHg, median (IQR)	212.5 (186.0-229.5)	200.0 (175.8-231.0)	219.0 (194.8-228.3)	0.31#
dBP on admission, mmHg, median (IQR)	122.5 (112.0-141.3)	121.5 (107.5-144.8)	123.0 (115.3-136.5)	0.89#
GCS on admission, median (IQR)	14 (10-15)	14.0 (10.0-14.8)	14.0 (10.3-15.0)	0.89#
Hematoma volume, mL, median (IQR)	9.4 (3.6-19.7)	9.7 (5.3-33.2)	8.0 (3.5-17.2)	0.32#
Hematoma site, n (%)				
Putamen	25 (41.7%)	14 (46.7%)	11 (36.7%)	
Thalamus	18 (30.0%)	8 (26.7%)	10 (33.3%)	
Cerebellum	7 (11.7%)	3 (10.0%)	4 (13.3%)	
Subcortical	6 (10.0%)	3 (10.0%)	3 (10.0%)	
Brainstem	4 (6.7%)	2 (6.7%)	2 (6.7%)	
Treatment characteristics and outcomes				
Surgical procedures, n (%)	17 (28.3%)	9 (30.0%)	8 (26.7%)	1.00†
Length of IV antihypertensives, hours, median (IQR)	26.8 (10.5-53.6)	33.2 (11.3-62.6)	23.6 (9.50-45.6)	0.44#
Integrated quantity of IV antihypertensives, γ×hours, median (IQR)	47.2 (30.3-144.4)	54.8 (31.6-146.2)	42.6 (30.2-126.7)	0.56#
Expansion of hematoma, n (%)	3 (5.0%)	2 (6.7%)	1 (3.3%)	1.00†
Hospital length of stay, days, median (IQR)	19.5 (14.8-28.0)	17.5 (14.3-22.0)	21.0 (16.3-30.0)	0.32#
ICU length of stay, days, median (IQR)	3.0 (2.0-7.0)	3.0 (2.0-7.0)	4.0 (2.3-6.8)	0.52#
Hypotension, n (%)	3 (5.0%)	1 (3.3%)	2 (6.7%)	1.00†
Acute kidney injury, n (%)	2 (3.3%)	2 (6.7%)	0 (0%)	0.49†
Hyperkalemia, n (%)	1 (1.7%)	0 (0%)	1 (3.3%)	1.00†
Time to antihypertensive regimen stabilization, hours, mean (SD)	131.4 (83.4)	119.6 (75.8)	143.3 (90.1)	0.28*
Scheduled oral antihypertensives when stabilized, n, median (IQR)	2.4 (2.0-3.0)	2.0 (2.0-2.0)	2.0 (2.0-3.0)	0.03#
sBP when stabilized, mmHg, median (IQR)	133.0 (129.5-136.0)	130.0 (125.3-136.0)	133.0 (132.0-136.0)	0.29#
dBP when stabilized, mmHg, median (IQR)	78.0 (68.0-89.3)	78.0 (68.5-89.5)	79.0 (69.0-88.8)	0.95#
mRS at discharge, median (IQR)	2.8 (1.8-4.0)	3.5 (2.0-4.0)	2.0 (1.0-4.0)	0.11#

Time to antihypertensive regimen stabilization (time to completion of regimen adjustment)

The mean time to completion of antihypertensive regimen adjustment was 119.6 hours in the ARNI group and 143.3 hours in the non-ARNI group. In univariate analysis, the difference was not statistically significant (p = 0.275; Table 1). In multivariable linear regression analysis (Table 2), ARNI use was independently associated with a shorter time to regimen stabilization (p = 0.045), while hematoma volume was also a significant predictor (p < 0.001).

**Table 2:** Multiple linear regression analysis of the outcomes (n = 60). A p-value ≤ 0.05 (bold) was considered statistically significant. B, partial regression coefficient; CI, confidence interval; SE, standard error; sBP, systolic blood pressure; ARNI, angiotensin receptor-neprilysin inhibitor; mRS, modified Rankin Scale. “ARNI group” is coded as 1 = ARNI and 0 = non-ARNI.

Factor	B	95% CI	SE	t	P-value
Time to antihypertensive regimen stabilization					
Age (years)	0.767	-1.003 to 2.538	0.883	0.869	0.389
Female	-20.405	-67.184 to 26.375	23.333	-0.875	0.386
sBP on admission (mmHg)	0.421	-0.139 to 0.982	0.280	1.507	0.138
Hematoma volume (mL)	1.585	0.796 to 2.373	0.393	4.029	<0.001
ARNI group	-40.427	-79.833 to 1.021	19.655	-2.057	0.045
Number of scheduled oral antihypertensives at regimen stabilization					
Age (years)	-0.001	-0.017 to 0.013	0.007	-0.211	0.834
Female	-0.179	-0.585 to 0.228	0.203	-0.880	0.383
sBP on admission (mmHg)	0.002	-0.002 to 0.007	0.002	0.962	0.340
Hematoma volume (mL)	0.005	-0.001 to 0.012	0.003	1.493	0.141
ARNI group	-0.483	-0.825 to -0.140	0.171	-2.823	0.006
mRS at discharge					
Age (years)	0.040	0.003 to 0.077	0.019	2.156	0.036
Female	-0.561	-1.546 to 0.424	0.491	-1.142	0.259
sBP on admission (mmHg)	-0.0002	-0.012 to 0.012	0.005	-0.032	0.975
Hematoma volume (mL)	0.029	0.012 to 0.046	0.008	3.512	<0.001
ARNI group	0.353	-0.476 to 1.183	0.414	0.854	0.397

Number of scheduled oral antihypertensives at regimen stabilization

**Figure 2:**
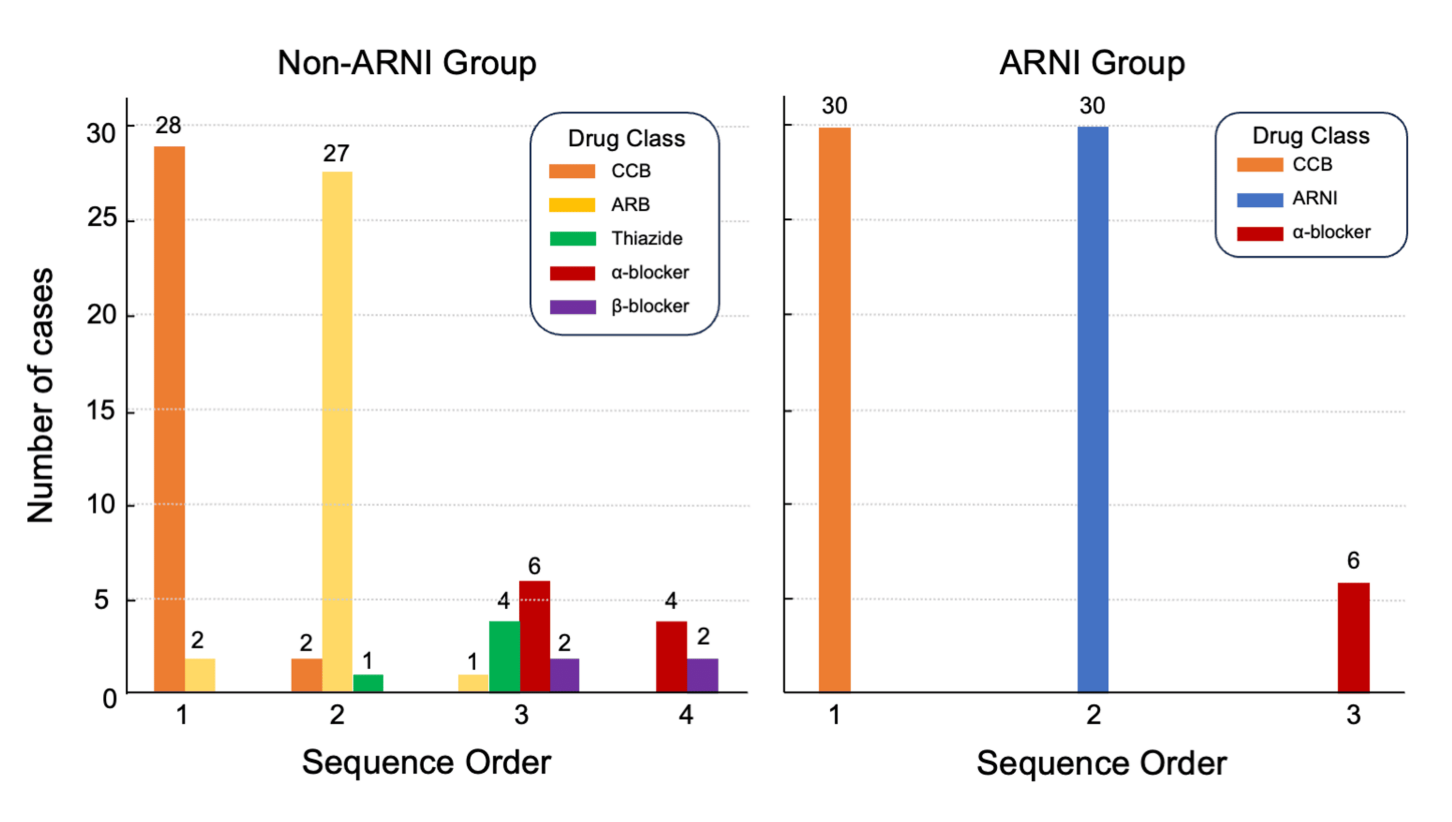
Initiation count by drug class and sequence order. In the non-ARNI group, first-line therapy was mainly a CCB and second-line predominantly an ARB; later lines included α-blockers, thiazides, and β-blockers. In the ARNI group, all patients received CCB first and ARNI second; α-blockers were added third in a minority. Counts are shown above bars. ARNI, angiotensin receptor-neprilysin inhibitor; CCB, calcium channel blocker; ARB, angiotensin II receptor blocker.

Figure 2 summarizes the sequence and class of scheduled oral antihypertensives. In the non-ARNI group, calcium channel blockers (CCBs) were most used as first-line therapy, with an angiotensin II receptor blocker (ARB) frequently added as second-line; subsequent additions included thiazide diuretics, α-blockers, and β-blockers. In the ARNI group, all patients received CCBs as first-line therapy, ARNI as second-line, and an α-blocker as third-line when needed. At regimen stabilization, the number of scheduled oral antihypertensive agents was lower in the ARNI group than in the non-ARNI group (p = 0.03; Table 1). In multivariable analysis (Table 2), ARNI use was independently associated with fewer scheduled agents (p = 0.006), with no other covariates showing significant associations.

Functional outcome at discharge

The mRS scores at discharge did not differ significantly between the ARNI and non-ARNI groups in the univariate analysis (p = 0.11; Table 1). In the multivariate linear regression analysis (Table 2), hematoma volume (p < 0.001) and age (p = 0.036) were associated with worse discharge mRS, whereas ARNI use was not significantly associated with discharge mRS (p = 0.397).

## Discussion

In this single-center retrospective cohort study of acute ICH patients with untreated refractory hypertension, introduction of ARNI as a scheduled second-line oral antihypertensive was associated with a shorter time to completion of antihypertensive regimen adjustment and a reduced number of scheduled oral antihypertensives required to achieve a stable oral regimen. Specifically, the unadjusted group difference in time to regimen stabilization was not statistically significant; however, after adjustment for clinically relevant covariates, particularly hematoma volume, ARNI use remained independently associated with a shorter time to regimen stabilization and fewer scheduled oral agents, suggesting that a CCB plus ARNI combination may facilitate earlier completion of antihypertensive regimen adjustment in routine clinical practice. This endpoint reflects completion of the antihypertensive regimen adjustment rather than physiological blood pressure stability.

ARNI is a combination drug comprising an angiotensin II type 1 receptor antagonist (valsartan) and a neprilysin inhibitor (sacubitril). It was first approved as an effective agent for heart failure owing to its superior efficacy compared to enalapril [13]; nonetheless, recently, its usefulness as an antihypertensive drug has also been recognized. ARNI lowers systolic and diastolic blood pressure more than the ARB alone dose, with evaluations conducted two months after initiation in patients with mild-to-moderate hypertension [14,15]. Per other reports, antihypertensive therapy by ARNI is beneficial in patients with refractory hypertension unresponsive to other agents [7,16], older patients with atherosclerotic disease [17], and in long-term management [18]. Nevertheless, evidence of early effects after initiation and stroke-related outcomes remains limited.

Per previous studies, such as the Intensive Blood Pressure Reduction in Acute Cerebral Hemorrhage Trial (INTERACT) 2 [6,7], various guidelines [1,19,20] recommend lowering blood pressure to sBP < 140 mmHg as early as possible during the acute phase of ICH and maintaining it for at least seven days. Recently, the usefulness of acute care management based on goal-directed care bundles has been reported from the perspective of functional outcomes (INTERACT3) [21], and early strict reduction of sBP (target value: <140 mmHg) has been incorporated into the core of this approach. Furthermore, suppressing blood pressure variability may be useful for improving functional prognosis [4,22]. Nonetheless, current guidelines provide limited guidance on the optimal choice and sequencing of oral agents when persistent hypertension continues after initial intravenous therapy. ARNI rapidly exerts its effects after administration, and its duration of action permits once-daily dosing [23,24]. ARNI reportedly exerts a stronger antihypertensive effect compared to ARB alone throughout the 24 hours, including at night and in the early morning [14,25], and may be a suitable antihypertensive drug for the acute phase of cerebral hemorrhage.

The results of our study have several clinical implications. A reduced number of scheduled oral antihypertensives provides valuable insights into the complexity of hypertension management and potential long-term treatment needs. Additionally, the use of fewer medications for blood pressure management can contribute to a decreased nursing workload, and a simplified medication regimen reduces the risk of medication errors and adverse drug interactions. Therefore, a second-line oral option with predictable and sustained blood pressure-lowering effects may be beneficial from an operational standpoint, even if downstream functional outcomes are not immediately improved in a modest-sized cohort.

Several limitations should be emphasized. First, the retrospective single-center design and use of historical controls raise concerns about selection bias and temporal confounding. Second, the sample size was modest, and confidence intervals for adjusted effects were wide, leaving the possibility of effect overestimation. Third, our primary endpoint reflected completion of antihypertensive regimen adjustment rather than direct physiological indices of blood pressure stability (e.g., time in target range or blood pressure variability). Detailed blood pressure variability metrics were not available due to the retrospective design and were therefore not evaluated. Fourth, although regimen stabilization may reflect clinically meaningful workflow and treatment simplification, the study was not powered to detect differences in clinical outcomes, and discharge mRS did not differ between groups. Fifth, the study population had relatively high functional status and small hematoma volume, which may limit the generalizability of our findings. Future studies should incorporate direct measures of blood pressure stability, evaluate downstream workflow-related outcomes (e.g., time to rehabilitation initiation, transfer to step-down/rehabilitation facilities), and confirm findings in larger multicenter cohorts.

## Conclusions

In acute ICH patients with untreated refractory hypertension, ARNI use as a scheduled second-line oral antihypertensive was associated with earlier completion of antihypertensive regimen adjustment and fewer scheduled oral antihypertensives. These results support the feasibility of incorporating ARNI into goal-directed acute blood pressure management strategies, while highlighting the need for confirmatory studies with physiologic blood pressure stability metrics and longer-term outcomes.
