# Correction: Sacubitril/Valsartan for Refractory Hypertension in Acute Intracerebral Hemorrhage: A Single-Center Retrospective Study

**DOI:** 10.7759/cureus.c447

**Published:** 2026-06-05

**Authors:** Koki Ito, Shinsuke Sato, Akitsugu Kawashima, Takakazu Kawamata, Yasunari Niimi

**Affiliations:** 1 Department of Neurosurgery, St. Luke’s International Hospital, Tokyo, JPN; 2 Department of Neurosurgery, Tokyo Women’s Medical University, Tokyo, JPN; 3 Department of Neuroendovascular Therapy, St. Luke’s International Hospital, Tokyo, JPN

Line 24 of the Materials and Methods section has been corrected to: The initial ARNI dose was sacubitril/valsartan 49 mg/51 mg or 97 mg/103 mg once daily (total daily dose: 100 or 200 mg), up-titrated according to blood pressure control to a maximum of 194 mg/206 mg once daily (total daily dose: 400 mg). The earlier version of this article had reported a dose of twice daily.

